# Immersive Virtual Reality Avatars for Embodiment Illusions in People With Mild to Borderline Intellectual Disability: User-Centered Development and Feasibility Study

**DOI:** 10.2196/39966

**Published:** 2022-12-07

**Authors:** Simon Langener, Randy Klaassen, Joanne VanDerNagel, Dirk Heylen

**Affiliations:** 1 Department of Human Media Interaction University of Twente Enschede Netherlands; 2 Centre for Addiction and Intellectual Disability Tactus Addiction Care Enschede Netherlands; 3 Nijmegen Institute for Scientist-Practitioners in Addiction Radboud University Nijmegen Netherlands

**Keywords:** virtual reality, VR, embodiment, avatar, embodied learning, body-centered, intellectual disability, addiction, user-centered design

## Abstract

**Background:**

Immersive virtual reality (IVR) has been investigated as a tool for treating psychiatric conditions. In particular, the practical nature of IVR, by offering a doing instead of talking approach, could support people who do not benefit from existing treatments. Hence, people with mild to borderline intellectual disability (MBID; IQ=50-85) might profit particularly from IVR therapies, for instance, to circumvent issues in understanding relevant concepts and interrelations. In this context, immersing the user into a virtual body (ie, avatar) appears promising for enhancing learning (eg, by changing perspectives) and usability (eg, natural interactions). However, design requirements, immersion procedures, and proof of concept of such embodiment illusion (ie, substituting the real body with a virtual one) have not been explored in this group.

**Objective:**

Our study aimed to establish design guidelines for IVR embodiment illusions in people with MBID. We explored 3 factors to induce the illusion, by testing the avatar’s appearance, locomotion using IVR controllers, and virtual object manipulation. Furthermore, we report on the feasibility to induce the embodiment illusion and provide procedural guidance.

**Methods:**

We conducted a user-centered study with 29 end users in care facilities, to investigate the avatar’s appearance, controller-based locomotion (ie, teleport, joystick, or hybrid), and object manipulation. Overall, 3 iterations were conducted using semistructured interviews to explore design factors to induce embodiment illusions in our group. To further understand the influence of interactions on the illusion, we measured the sense of embodiment (SoE) during 5 interaction tasks.

**Results:**

IVR embodiment illusions can be induced in adults with MBID. To induce the illusion, having a high degree of control over the body outweighed avatar customization, despite the participants’ desire to replicate their own body image. Similarly, the highest SoE was measured during object manipulation tasks, which required a combination of (virtual) locomotion and object manipulation behavior. Notably, interactions that are implausible (eg, teleport and occlusions when grabbing) showed a negative influence on SoE. In contrast, implementing artificial interaction aids into the IVR avatar’s hands (ie, for user interfaces) did not diminish the illusion, presuming that the control was unimpaired. Nonetheless, embodiment illusions showed a tedious and complex need for (control) habituation (eg, motion sickness), possibly hindering uptake in practice.

**Conclusions:**

Balancing the embodiment immersion by focusing on interaction habituation (eg, controller-based locomotion) and lowering customization effort seems crucial to achieve both high SoE and usability for people with MBID. Hence, future studies should investigate the requirements for natural IVR avatar interactions by using multisensory integrations for the virtual body (eg, animations, physics-based collision, and touch) and other interaction techniques (eg, hand tracking and redirected walking). In addition, procedures and use for learning should be explored for tailored mental health therapies in people with MBID.

## Introduction

### Background

Immersive virtual reality (IVR) has been investigated as a treatment tool for a variety of psychiatric disorders, for instance, in people with psychosis, addictive disorders, and eating disorders [1,2]. However, so far, the clinical effectiveness has only been proven in anxiety disorders, as (randomized) controlled trials in other mental illnesses are still required. However, the practical nature as *doing instead of talking* approach makes IVR therapy appealing for people who do not profit from existing treatments, such as people with mild to borderline intellectual disability (MBID; IQ=50-85). People with MBID constitute a diverse group with low intellectual and adaptive capabilities (eg, problems in planning, problem solving, abstract thinking, and judgment), which negatively affects the development of essential skills for independent living. By using the term MBID, we combine the groups mild intellectual disability (IQ=50-69) and borderline intellectual functioning (IQ=70-85), as they often encounter similar challenges in life, for instance, regarding mental health treatments [3-5]. Previous studies suggest that IVR could help to reduce learning barriers, by making abstract concepts and interrelations graspable [6-8], bypassing the need for a disembedded thinking [9]. In addition, IVR for the MBID group could transfer tangible content for an active rather than passive learning [10], thus evading the need for excessive language use and fostering skill acquisition by making mistakes [9]. However, applications of IVR and knowledge about requirements in MBID remain low [11], as few researchers have explored the interaction design using state-of-the-art hardware [12-15]. However, turning with one’s own body and interacting via hand-based manipulations were found to benefit usability. Hence, implementing interaction techniques that provide a user experience similar to that of real life seems vital, for instance, by immersion into a virtual body. This *embodiment illusion* could facilitate life-like behavior and therefore improve the access to IVR for our group [16].

Embodiment illusions in IVR allow us to substitute the real body with a virtual body or certain body parts, such as arms or hands [17-19]. The phenomenon is often assessed by the *sense of embodiment* (SoE) toward the virtual body (ie, *avatar*) [19,20]. The “SoE toward a body B is the sense that emerges when B’s properties are processed as if they were the properties of one’s own biological body.” (p375) [19]. Hence, the embodiment illusion is induced through 3 main factors: sense of self-location, agency, and ownership [19,21]. The *sense of self-location* refers to the feeling of being inside the body [19]; the *sense of agency* comprises the “global motor control, including the subjective experience of action, control, intention, motor selection and the conscious experience of will” (p7) [22]; and the *sense of body ownership* involves the self-attribution to the avatar [19]. However, so far, the significance of each factor for the illusion remains ambiguous [19]. Nonetheless, illusions of virtual body ownership (IVBO) were found to influence the user’s attitudes and behavior [23], which makes them promising for enhancing therapy outcomes in groups that hardly benefit from cognitively demanding paradigms, such as people with MBID.

Previous findings in people devoid of MBID showed that embodying a Black avatar can reduce racial bias and that embodying a child can influence implicit attitudes and object size perception in IVR [24,25]. Both refer to the *proteus effect*, derived from the Greek myth of a *shape shifter*, describing the phenomenon that we (humans) tend to change our beliefs and behavior based on our (digital) self-representations [16]. For instance, as empathy training, body swapping was used to present power relationships between offender and victim in sexual harassment, subsequently reducing conformity in social pressure scenarios [26]. In addition to such implicit approaches, explicit learning could be applied, for instance, psychomotor addiction therapy with a focus on bodily signals (eg, cravings) [7], by using the virtual body as a multimodal feedback system. However, despite various studies that report on the design requirements for such IVBO, no study has focused on people with MBID. Hence, this study aimed to design IVR avatars for embodiment illusions in individuals with MBID. As the spatial immersion into the IVR avatar and implementation of plausible actions (eg, controls) can evoke realistic behaviors [16], we decided to look into three important components for embodiment illusions: (1) avatar appearance, (2) controller-based locomotion, and (3) object manipulation.

### Related Studies

#### Overview

In the following sections, we examine related studies concerning (1) avatar immersion, (2) controller-based locomotion, and (3) object manipulation. Given the lack of studies in our target group, we report on the existing evidence in non-MBID samples to identify crucial factors for our initial prototype and immersion design. We conclude the *Introduction* section with a summary of potential benefits and barriers of embodiment illusions for people with MBID and our research questions. Then, we describe our user-centered design method with 3 iterations and report the results per iteration. Subsequently, we discuss relevant factors in the context of previous studies, limitations, and directions for future research. Finally, we conclude our paper with a summary of our contribution to the field.

#### Immersion Into IVR Avatars for IVBO

Several factors contributing to IVR avatar immersion have been investigated to influence SoE, such as the point of view (POV; ie, the perspective), body appearance, control, and haptics (ie, the experience of touch) [19]. For instance, an egocentric POV has been shown to reliably induce the *sense of self-location* [27], whereas a third-person perspective tends to lower it [28,29]. The *sense of agency* is induced through the experienced control of the virtual body [19], influenced by the visuomotor congruence between the real body and avatar [30-32], whereas incongruences tend to lower it [33,34]. Finally, the *sense of ownership* is influenced by body appearance and has been induced through avatar models with different degrees of anthropomorphism [35]. However, despite the possibilities to feel ownership toward avatars that differ from oneself in terms of gender and morphological characteristics [36,37], matching gender, skin tone, and clothes can boost IVBO [38,39]. However, the SoE factors cannot be considered to be isolated from each other, as interrelations have been identified [40], such as influence of appearance on agency [41] and control and haptics on ownership [42-44]. Here, recent findings showed that primarily visuoproprioceptive congruence contributes to the *sense of agency* and *ownership* and better task performance [32]. Moreover, Fribourg et al [40] explored the user preferences for 3 vital factors (ie, POV, control, and appearance), showing the need for an egocentric perspective and high motor control to outweigh the avatar’s appearance. However, these findings seemed to be task dependent, with POV being relevant for locomotion and avatar appearance when manipulating (virtual) objects using the upper body [40,41,45].

#### Controller-Based Locomotion for IVR Avatars

Controller-based locomotion, as an essential component of immersion into IVR avatars, can be divided into physical and artificial approaches [46]. Physical techniques can be more intuitive (ie, room scale); however, an intensive bodily involvement and unnaturalness (eg, walking in place) may cause the opposite effect. In contrast, artificial techniques (eg, teleport and joystick) tend to increase the cognitive workload and are prone to cause cybersickness (ie, motion sickness) [46,47]. As space for natural locomotion is often limited, adding artificial techniques of continuous (eg, controller-based) or noncontinuous (eg, teleport-based) nature could form a viable solution [13]. Continuous approaches are preferred in open settings, whereas noncontinuous approaches are widely used owing to their user-friendliness [46]. However, few studies have explored virtual locomotion in combination with IVR avatars, showing its influences on task performance and obstacle avoidance [48-50]. The virtual body can improve walking behavior in IVR, with fewer collisions, more precise paths using a realistic avatar [51], and more natural behavior [52]. Here, using walking animations that mimicked natural behavior were preferred over the user’s real motions (ie, walking in place); however, this could lead to unintended steps [53]. Nevertheless, few studies have examined the effects of virtual locomotion on the SoE factors. Dewez et al [50] compared natural walking, walking in place, and virtual steering and found a similar SoE, with equal performance with or without an avatar. Consistent with previous findings, movement incongruences between the user and avatar animation did not break the embodiment illusion [28].

#### Object Interaction for IVR Avatars

Similar to avatar immersion and locomotion design, interacting with objects and user interfaces (UIs) are essential components for immersive self-representations. Here, using avatars influences the interaction with objects and vice versa [45]. The alignment of this reciprocity to design for both high SoE and usability remains understudied in the current literature, especially when combined with artificial locomotion. Previous studies that used IVR avatars during interaction tasks reported performance enhancements over controllers or virtual hands [54], independent of the model’s human-likeness, when comparing a realistic avatar with a generic or robot appearance [55,56]. However, as spatial biases were found in IVR [57], the body may operate as a reference frame [58], as the object size perception can be altered when using avatars [59]. Here, avatars can produce occlusions during interactions, which can affect the usability negatively, especially when using more anthropomorphic models [41,60]. However, using congruent body feedback could circumvent this issue, considering that haptics (eg, self-touch) benefits SoE and manipulation performance [32,61,62]. Finally, identified usability barriers are objects that are out of reach or placed low. A solution devoid of altering the avatar or breaking the embodiment illusion could be artificial interactions (eg, raycasting) implemented into the avatar’s hands [45,63], allowing interaction with objects without substantial bodily movements.

### Goal of This Study

In summary, embodying an IVR avatar may improve usability [51], spatial awareness [6,45], and (self) presence [31,64]; however, adverse effects can occur owing to an increase in complexity [60]. Nonetheless, so far, design requirements for such IVR avatars and the feasibility to induce IVBO have not been explored in people with MBID. Despite the proposed use for the treatment of psychiatric disorders, IVR applications focusing on life skills (eg, public transport and grocery shopping) [12,13], vocational training [65], and (motor) rehabilitation for MBID could be beneficial [14,66]. The virtual body may reduce the cognitive workload and enables novel forms of visuomotor feedback, for instance, to support the problematic hand-eye coordination of individuals with intellectual disability in IVR [67]. So far, IVBO was mostly investigated in controlled laboratory settings with motion-capture systems or several body trackers to congruently map the user’s bodily movements onto the virtual body. Although these systems seem to provide the highest control, they lack consumer-friendliness to enter care institutions, given the high costs and difficulty in using the equipment. Here, solutions based on inverse kinematics (IK) that use 3-point tracking of the head-mounted display (HMD) and related controllers could provide an alternative, as most interactions focus on the upper body [40]. Hence, we aimed to explore guidelines for such IVBO in people with MBID, by conducting a user-centered development based on three factors that contribute to functional and plausible actions [16]:

1. How to design IVR avatars for IVBO in people with MBID?
  - How to design a virtual embodiment illusion for people with MBID based on IK?
  - How to design a controller-based locomotion technique for people with MBID?
  - How to design a controller-based (object) manipulation for people with MBID?
2. To what extent do participants experience SoE during the examined interaction task?
3. To what extent do participants experience a sense of presence (SoP) in the immersive virtual environment (IVE)?

## Methods

### Research Design

We conducted a user-centered design approach to explore the 3 factors for IVR avatar immersion (ie, avatar, controller-based locomotion, and manipulation), initial feasibility of procedures, and proof of concept of IVBO in people with MBID. For this, we developed an IVR avatar prototype to identify design recommendations for IVBO using 3 consecutive iterations with end users in Dutch care facilities. Throughout these iterations, we refined the IVR avatar system and immersion procedure according to the participants’ needs. Hence, in this study, we established design recommendations for the 3 components and explored the SoE levels and SoP in the IVE to support others in creating accessible IVR avatars.

### Participants

In total, 29 adults with MBID were recruited through convenience sampling by local therapists from an addiction clinic for individuals with MBID and Dutch care facility for people with MBID. Exclusion criteria included having a history of migraine, epilepsy, visual or motor impairment, or severe mental disorder (eg, schizophrenia, psychosis, or active substance use disorder); susceptibility to COVID-19; proneness to motion sickness; or inability to wear the HMD.

### Interaction System

We built the interaction system with consumer hardware and available software. The game engine Unity3D (version 2019.4 LTS; Unity Technologies) was used with the Mecanim IK and the eXtended Reality interaction toolkit (preview; version 0.94) packages to develop an IVBO based on 3-point tracking of the HMD and the related 2 controllers. We implemented the three identified components for IVR avatars: (1) customizable avatar, (2) controller-based locomotion, and (3) object manipulation.

**Figure 1:**
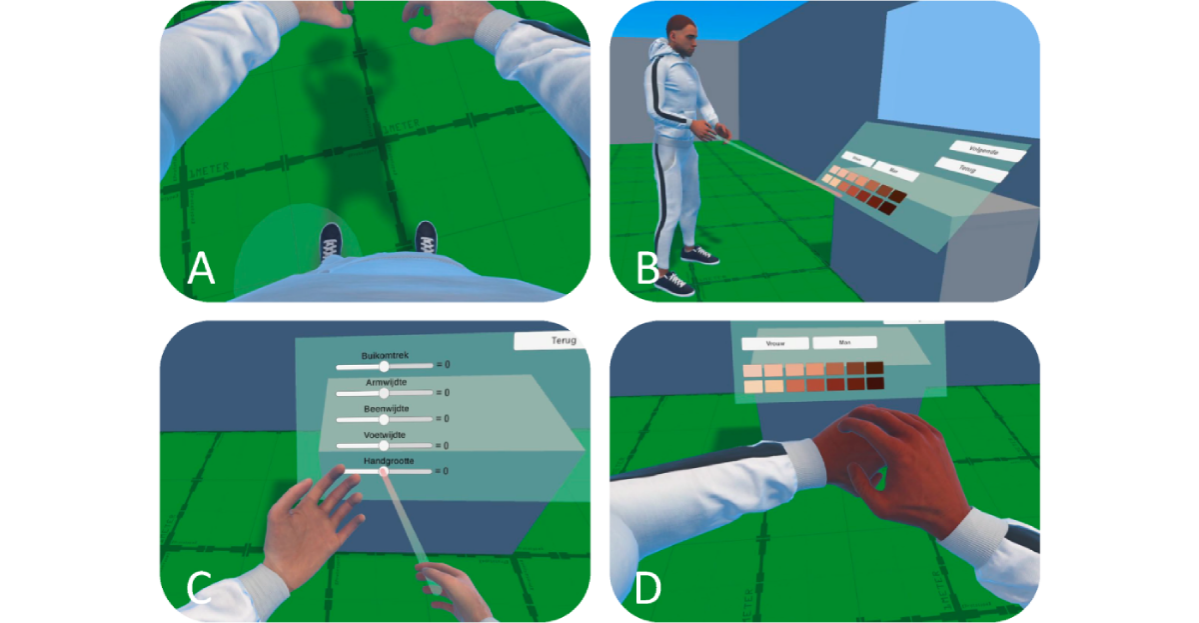
The customizable immersive virtual reality avatar: (A) egocentric perspective, (B) raycast interaction with user interface, (C) customization of body dimensions, and (D) touching the nondominant hand with the dominant one.

The customizable IVR avatar component (Figure 1) included an egocentric embodiment illusion. The participants were able to enter their height and arm dimensions (ie, by going into the T-pose), customize their gender (woman or man), and select a skin tone.

**Figure 2:**
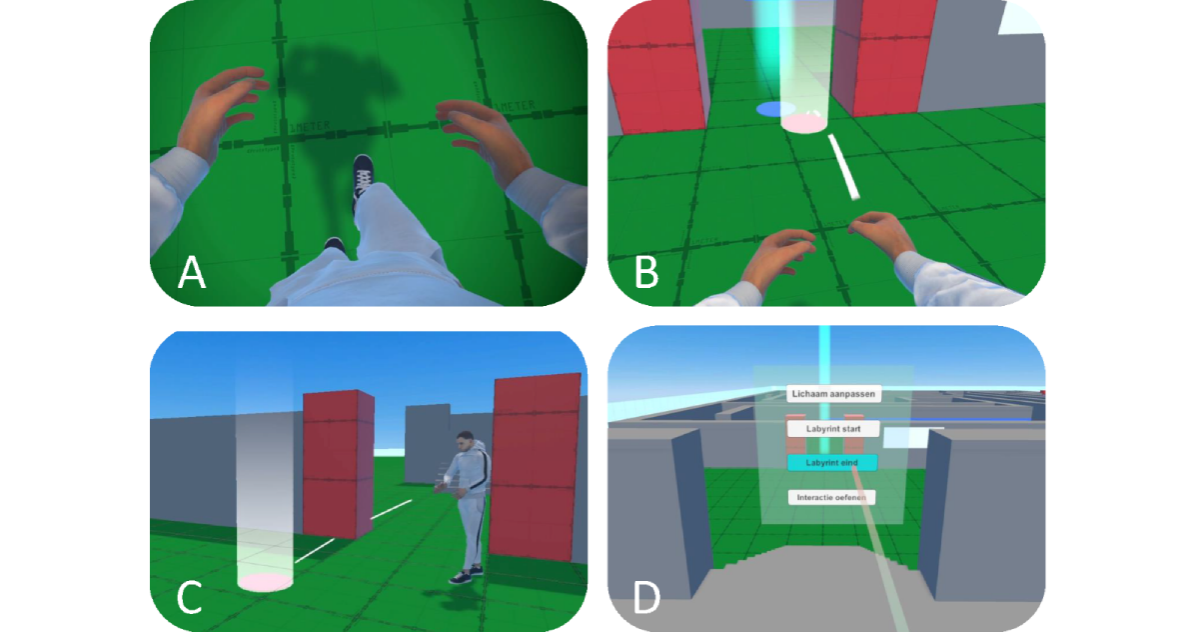
The controller-based locomotion: (A) joystick locomotion with walking animation, (B) teleportation locomotion using raycasting with a projectile curve, (C) hybrid locomotion (A+B), and (D) teleport travel to interaction tasks.

The controller-based locomotion component (Figure 2) involved a visuomotor experience of moving in the IVE, divided into physical and artificial approaches. The physical approach comprised basic room-scale locomotion (2×2 m), with walking animation when moving. Overall, 3 artificial locomotion approaches were implemented — divided into joystick locomotion with 45° snap turn and walking animation, teleportation locomotion using raycasting with a projectile curve, and a combination of both (hybrid). Haptics were used at the beginning and when executing teleportation by using the controller’s vibration motors. Furthermore, a *teleport travel* technique that enables the transition to the different interaction contexts by using a screen-space UI was implemented [68], which followed the user’s rotation on the y-axis when holding the A button.

**Figure 3:**
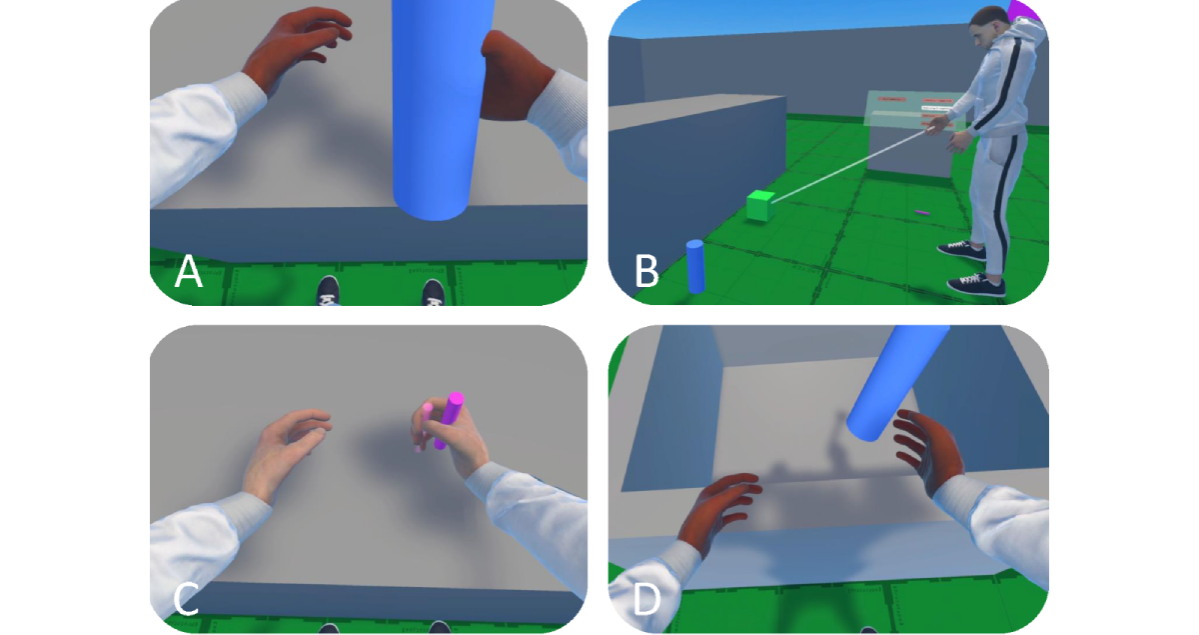
The controller-based object manipulation (A) grabbing objects on the "table", (B) picking-up objects using sphere-casting, (C) placement of objects at cued locations, and (D) throwing objects into a box.

The (object) manipulation component (Figure 3) included a synchronous visuomotor experience to grab, pick up from low areas, place, and throw virtual objects. We used hand animations for grabbing (grip button) and pinching (trigger button), including haptics, when grabbing and releasing the objects by using the controller’s vibration motors. Raycasting on both hands was implemented to pick up objects that are placed low or out of reach and interact with UIs.

### Hardware and IVE

We used an Oculus Quest HMD with 6 df, 1440×1600 pixels per eye, 72 Hz refresh rate, and 90° field of view (FOV); touch controllers; and a compatible IVR-laptop (Intel Core i7 9750H central processing unit; 16 GB RAM; NVIDIA GeForce RTX 2060) with Oculus Link (beta; USB 3.1 cable).

The IVE encompassed an open-world mechanic (200×200 m) to evaluate the system’s components. In the first room setting (15×15×2.5 m), the participants customized the IVR avatar. In the second room, artificial locomotion techniques were evaluated by completing a maze (50×50×2.3 m) with 4 destinations and obstacles to provoke different user movements. On the basis of common game design, we used a vantage point to support spatial understanding and reduce unease. Further, destinations were cued using light beams of different color, with matching leading lines on the walls [69]. In the third context (15×15×2.5 m), we evaluated 4 basic object manipulation tasks to ensure a broad coverage of possible IVR interactions. For this, we used 3 different objects (ie, large cylinder, cube, and small cylinder) to grab and release each object (Figure 3A), pick up the object from the ground (Figure 3B), place objects at another location (Figure 3C), and throw all objects into a box (Figure 3D). The corresponding task completion was detected by the system automatically (eg, object grabbed and released), allowing users to transition to the next task. Participants used object-spaced UIs with low hierarchy to customize the avatar, select locomotion approaches, and control interaction tasks. A plain design was used to reduce bias, and we implemented landmarks (pink in color) to aid user’s orientation in the IVE.

### Measures

A semistructured interview (Multimedia Appendix 1) was conducted after each of the 5 interaction tasks: IVR avatar customization, teleport, joystick, hybrid locomotion, and object manipulation. For the IVR avatar, we aimed to explore the first impression, customization choices, usability issues, ownership perception, and points for improvement. For locomotion, we explored the first impression, usability issues, and impression of the body during locomotion. The questions for all locomotion techniques were identical. Regarding object manipulation, we asked for the first impression, usability issues, enjoyable aspects, and perception of the body during interaction. Finally, we evaluated the impressions and usability issues concerning IVE, UI interactions, and intentions for using the system.

SoE was assessed using an adapted version of the Virtual Embodiment Questionnaire (VEQ) [20]. The VEQ is a 12-item questionnaire assessing the SoE subscales ownership (Cronbach α=.78), agency (Cronbach α=.76), and change (Cronbach α=.77). In addition, 3-items assessing the sense of self-location were adapted to this research context to extend VEQ [21,30]. Scores for each item ranged from 1 (“strongly disagree”) to 7 (“strongly agree”).

SoP was assessed using an adapted version of the Igroup Presence Questionnaire (IPQ) [70]. The IPQ (Cronbach α=.85) is a 14-item questionnaire assessing the SoP subscales general presence, spatial presence (Cronbach α=.80), involvement (Cronbach α=.76), and experienced realism (Cronbach α=.68). Scores for each item ranged from 1 (“strongly disagree”) to 7 (“strongly agree”).

Considering the needs of people with MBID, we adapted questionnaires in language and complexity (by using plain Dutch language) with an expert from the field. This implies that questions asking for 2 different concepts were reduced to one; for example, “I felt like the form or appearance of my own body had changed” was changed to “I felt like the form of my own body had changed” [20]. In addition, complex formulations were simplified; for example, “Somehow I felt that the virtual world surrounded me” was changed to “I felt that the virtual world surrounded me” [70].

### Ethics Approval

Ethics approval was obtained from the University of Twente’s ethics committee (RP 2020-164) and the care institution’s scientific board.

### Procedure

To comply with COVID-19 precautions, the researcher disinfected the materials and IVR apparatus before evaluation. Before evaluation, disinfection of hands and forearms was required, a medical mask was used by the researcher, and a distance of 1.5 m was maintained whenever possible.

Participants were welcomed and informed about the study procedure before starting the experiment to comply with the ethical principles in accordance with the Declaration of Helsinki. The researcher explained the IVR technology, controls, and possible adverse effects. After informed consent was obtained, the participants were immersed into the IVE. In addition to visual in-game cues, verbal instructions were used to guide the user through the procedure.

Before assessing the prototype version, participants found themselves in the customization room with controller models enabled, but deactivated avatar. First, users were asked to set the interpupillary distance using the HMD slider for proper vision. Then, a short acclimatization period was conducted to enhance spatial understanding, which includes the basic room-scale boundaries and locomotion. After the remaining questions were answered, the assessment of the different components was initiated.

The first task involved avatar customization (Figure 1). Participants were instructed to go into T-pose to conduct the scaling procedure, followed by the selection of gender and skin tone on the UI (Figure 1B), which enabled the avatar (Figure 1A). The participants were given a maximum of 5 minutes to explore the avatar, hand, and walking animations using room-scale locomotion. After approximately 3 minutes, the participants were asked to touch the nondominant hand by using the dominant one to explore the self-location of hands through a tactile sensation (Figure 1D). Then, users were asked to remove the HMD, so that the extended VEQ and dedicated semistructured interview questions can be assessed by the researcher. After completion, participants were asked to wear the HMD again to proceed to the next component.

The second task evaluated the controller-based locomotion techniques to move in the IVE (Figure 2). The participants were asked to *teleport travel* to the vantage point by using the related UI (Figure 2D). Then, participants enabled the predefined locomotion approach, that is, joystick (Figure 2A), teleport (Figure 2B), or hybrid (Figure 2C). Following an introduction to the technique, participants were asked to complete the maze. In case of severe motion sickness, participants were allowed to stop early to complete the remaining procedure. Upon completion, participants were asked to remove the HMD to assess the extended VEQ and corresponding interview questions. Then, end users were asked to wear the HMD again to evaluate the remaining locomotion techniques by following the same procedure in an overall counterbalanced manner.

The third task included different manipulations of 3 objects in a room setting (Figure 3). First, participants were asked to *teleport travel* to the locomotion UI to enable the preferred technique. Then, participants were instructed to *teleport travel* to the manipulation tasks and move to the interactables and related UI. The participants were instructed to (1) grab and release each object (Figure 3A), (2) pick up the object from the ground using raycasting (Figure 3B), (3) place objects at another location based on cues (Figure 3C), and (4) throw objects into a box (Figure 3D). After completion, the participants were again asked to remove the HMD and the extended VEQ and related interview questions were assessed.

Following the evaluation of the 3 avatar components, participants were asked to provide demographic information and to complete the IPQ and remaining interview questions. Finally, users were debriefed and encouraged to express remaining questions or concerns, and they were subsequently thoroughly answered. The participants received a small nonmonetary gift as a sign of gratitude (approximately €10 [US $10]).

### Data Analysis

Qualitative data were analyzed based on the thematic analysis approach by Braun and Clarke [71]. To account for the research design, we divided data sets from the iterations into three segments each: (1) avatar customization, (2) artificial locomotion, and (3) manipulation. In these segments, coding was applied to the data that were transcribed verbatim to identify themes by conducting a recursive process using Atlas.ti (version 9.1.4; ATLAS.ti GmbH). The coding process was continuously discussed among the researchers (ie, SL, JV, and RK). Quantitative data regarding the extended VEQ and IPQ subscales were described for each iteration and on an aggregated level. Descriptive analyses were conducted using RStudio (version 1.3.1093).

## Results

### Sample Description

Table 1 presents the sociodemographic characteristics and technological experience of the sample. Of the 29 participants, 5 participants (17%) terminated prematurely owing to severe motion sickness (3/5, 60%), anxiety (1/5, 20%), or use inability (1/5, 20%), resulting in missing experimental and demographic data. The remaining participants (24/29, 83%) had a mean age of 34.2 (SD 9.8) years, and most identified as male (23/24, 96%). The sample was equally composed from the 3 institutions (8/24, 33% from each) and included participants with borderline intellectual functioning (IQ=70-85; 13/24, 54%) and mild intellectual disability (IQ=50-69; 11/24, 46%). The technology experience with computers and videogames was rated high compared with virtual reality. The following sections describe the process that led to our final prototype and procedural considerations identified during the design process.

**Table 1:** Sociodemographic characteristics and technological experience of the sample.

Sample characteristics			Iteration 1 (n=6)		Iteration 2 (n=12)		Iteration 3 (n=6)		Full sample (n=24)
Age (years), mean (SD)			33.83 (9.24)		36 (8.77)		30.83 (12.78)		34.17 (9.77)
**Gender, n (%)**									
	Men	6 (100)		11 (92)		6 (100)		23 (96)	
	Women	0 (0)		0 (0)		0 (0)		0 (0)	
	Diverse	0 (0)		1 (8)		0 (0)		1 (4)	
**Intelligence, n (%)**									
	Borderline intellectual functioning	3 (50)		6 (50)		4 (67)		13 (54)	
	Mild intellectual disability	3 (50)		6 (50)		2 (33)		11 (46)	
**Institution, n (%)**									
	Addiction clinic	2 (33)		4 (33)		2 (33)		8 (33)	
	Care institution	2 (33)		4 (33)		2 (33)		8 (33)	
	Forensic addiction clinic	2 (33)		4 (33)		2 (33)		8 (33)	
**Technology experience^a^, mean (SD)**									
	Computer	5.83 (0.75)		5.50 (1.38)		5.17 (1.47)		5.50 (1.25)	
	Video games	4.17 (0.75)		5.83 (1.34)		6.17 (0.98)		5.50 (1.35)	
	Virtual reality	2.17 (1.47)		3.08 (1.88)		3.33 (2.66)		2.92 (1.98)	

^a^Technology experience was assessed using a 7-point Likert scale, ranging from 1 (“No experience”) to 7 (“A lot of experience”).

### Iterative Prototype Development

#### Findings From Iteration 1

##### Overview

Most participants (4/6, 67%) reported a positive first impression regarding the IVR avatar system. The scaling procedure was feasible; however, visual in-game instructions were lacking. Here, the avatar’s congruence with one’s own self-concept was essential, because users selected their own skin tone and gender, reporting on the desire to replicate their own body image:

I’m really a slim puppet now. In real life, I have a bit of a belly.

Participant 4

Furthermore, technical issues were described, such as unrealistic wrist movements and arm glitches and the absence of haptics (self-touch) properties:

You cannot grasp it, so it is not yours.

Participant 1

Regarding joystick locomotion, participants reported usability issues when using the 45° snap turn, further contributing to the prevalent cybersickness:

When you hit the wall, it gets really bad.

Participant 3

In contrast, cybersickness was absent during teleport locomotion:

The teleporting went better. I did feel better.

Participant 3

However, usability issues owing to the limited flexibility and own pace were described:

At one point it went too fast. Then it seems too easy, but then you have to take a step back.

Participant 5

In contrast to the joystick approach, during teleport use, participants missed the human-like walking illusion:

Seems like I just really walk, so to speak.

Participant 6

The hybrid approach (ie, joystick and teleport) showed no added value, as participants relied on their preferred technique. However, all approaches showed a need for control habituation and attention shift from avatar to task.

Finally, for (object) manipulation, the usability was rated positively; however, participants reported issues with raycasting, either because it was always enabled or because it was difficult to hit the objects on the ground. Owing to the lack of intuitiveness (“Normally you can bend down and grab.” [participant 2]) and haptics, a need for control habituation was essential:

Because it feels very different from when you’re actually grabbing something.

Participant 3

Of the 6 participants, 2 (33%) reported on interaction realism (“But you know, all the movements, the behavior is indeed real*.*” [participant 5]), whereas another participant missed realistic gabbing animations by using the virtual hands instead of the controller. The UI interaction showed good usability, despite the need for repositioning to use the object-spaced UIs.

##### Changes for Iteration 2

Regarding the IVR avatar, the snap turn was refined from 45° to 15° to remove usability issues and alleviate cybersickness. To improve manipulations, the grabbing attachment was changed to the avatar’s hands instead of the controller anchor, and raycast activations were reduced to objects below 50 cm. To enable further customization, alteration of the model’s body dimension (size of the arm, belly, leg, and feet) was implemented (Figure 1C). We also improved implementation issues that led to a smaller scaling of the model. Furthermore, we refined the accuracy of the hand IK targets for better proprioception. Finally, as the VEQ items seemed complex for our target group, we added 2 questions to the semistructured interview (Multimedia Appendix 1), asking for the sense of ownership after each interaction task and the perception of change after the evaluation procedure.

#### Findings From Iteration 2

##### Overview

Accordant with the first iteration, replicating one’s own body image was paramount, self-touch and physical collision were lacking, and some animation issues (ie, arm glitches and unrealistic leg movements) were reported. In contrast, participants reported on the clothing, either owing to the incongruence (“Because the suit I was wearing didn’t match with what I was wearing.” [participant 15]) or illusion of wearing the virtual clothes (“I was really convinced in my head, that I was wearing it today.” [participant 10]). Technical issues included a restricted view toward the lower body when bending (“When I look down like that, all of a sudden, I got a belly.” [participant 9]), which also hindered customization owing to occlusion. Moreover, hand size adjustments and customization aids (ie, presets) were lacking. Notably, the human-likeness was described as mostly positive; however, a user missed the haptics and described the uncanny valley:

I actually found that a bit creepy. Because your hands actually looked like real hands.

Participant 10

Regarding artificial locomotion, cybersickness and snap turn issues remained during joystick walking. Further usability remarks included inaccurate wall collisions with lacking haptics, inaccurate physics (ie, weight), unrealistic foot tilting (“So when I walked fast, my feet just shuffled*.*” [participant 17]), preferences for walking using room scale, and advanced movements (ie, running, jumping, and climbing). As in the previous iteration, participants missed the human-like walking illusion (“I did walk by myself but also didn’t*.*” [participant 13]) during teleport (“It was a little inhuman*.*” [participant 19]); however, some participants habituated:

At some point when you do figure it out, yes, then it will probably be a little easier.

Participant 11

Nonetheless, other usability problems, such as limited range, restricted mobility, fast pace resulting in errors, and activation issues, were described. The required bodily turning was perceived as ambiguous, with a participant suggesting the addition of a snap turn. Consistent with the previous iteration, users relied on their preferred technique for hybrid locomotion, and all techniques showed a need for control habituation and attention shift from body to task.

Finally, manipulation usability was rated positive; however, the need for habituation periods for movements and controls, that is, switching between locomotion and object manipulation, controller assignment (“What is where? A and B, joystick*.*” [participant 8]), and limited room-scale area (“If you had more space, you could just walk there*.*” [participant 9]), were described. As in the first iteration, hitting objects with raycasting was troublesome. Furthermore, a participant reported on missing haptics:

On the one hand, it feels very familiar and on the other, it’s unrealistic that I don’t feel.

Participant 10

Here, half of the participants (6/12, 50%) described the interactions as realistic; however, few participants mentioned the lack of grabbing realism and unrealistic physics or collision resulting in occlusion. Despite needing habituation, all participants (12/12, 100%) reported good usability regarding the UIs.

##### Changes for Iteration 3

For the third iteration, we simulated bending using backward placement of the virtual body, aiming to increase the visuoproprioceptive congruence for more natural behavior. Furthermore, we refined the accuracy of the IK targets and improved the smoothness of the body rotation, by including influences of the hand locations. Moreover, we removed arm scaling owing to the preponderant symmetric nature of human bodies and difficult scaling procedures. Notably, we deactivated the HMD’s energy saving option, because we discovered floating floor levels after reactivation, which we aimed to account for during the iteration. To improve object manipulation realism and remove usability issues, the *idling* hand animation was refined, and objects were picked up with spherecasts instead of raycasts (ie, *magnetic* toward the object). To allow further customization, we added an option for hand size adjustment to the related UI. Finally, we implemented a dynamic FOV reduction (ie, vignetting) to alleviate cybersickness during artificial locomotion.

#### Findings From Iteration 3

As in the previous iterations, replicating one’s own body image through customization was key. Participants described the avatar as human-like (*“*It looks real, and I also felt that I was touching my own hand.” [participant 24]) with congruent haptics, and 33% (2/6) of the participants justified customization choices (ie, skin tone) in contexts of social meaning:

And it’s not because I’m racist.

Participant 24

Similar to the previous iterations, minor technical issues such as an unrealistic wrist movement and imprecise bending of legs remained.

Regarding joystick locomotion, cybersickness and snap turn issues remained, but were reported to be less severe. Participants rated the embodiment as positive, reporting on the human-like walking illusion; however, a participant described an unrealistic foot tilting. Furthermore, the preference for turning using one’s own body, inaccurate wall collision, and attention shift to the task were described. Consistent with the previous iterations, cybersickness was absent during teleport locomotion; however, usability issues owing to the fast pace, activation issues, and turning using one’s own body remained:

I had to turn but I couldn’t walk.

Participant 22

In addition, control issues that rotate the user after teleporting were reported. However, the avatar was rated as positive, even though participants described an ambiguous human-likeness when teleporting and attention shift from avatar to task. Accordant with the other iterations, all approaches showed a need for control habituation, and the hybrid locomotion remained mostly unused.

Consistent with the previous iterations, participants reported positive manipulation usability despite the need for initial (control) habituation. Only 17% (1/6) of the participants mentioned selection issues when using the object-spaced UI, whereas another participant preferred the screen-spaced approach over the object-spaced one. Finally, the embodiment during manipulations was rated as positive and human-like, with a participant describing the feeling of haptics through the controller’s vibration motors (*“*When I grabbed something, I also felt a vibration through my hand...It really felt like I was holding something*.”* [participant 19]) and unused artificial locomotion owing to the immersion (*“*I forgot that I could also walk with my joystick*.*” [participant 19]).

### SoE Related to IVR Avatar Task

Table 2 shows the extended VEQ scores throughout our iterative development. The contextual differences indicate that the sense of ownership tends to increase with growing interaction capabilities, whereas the perception of change (in the perceived body schema) decreases. In contrast, the sense of self-location and agency scores remained relatively stable across measurements, with positive agency trends during interactions, whereas self-location feelings decreased. Interestingly, ownership and agency scores regarding teleport locomotion were lower than those in other active contexts, which matches the qualitative data.

The qualitative data indicate that IVBO was dependent on habituation (*“*Just a matter of getting used to it*.*” [participant 3]), sense of agency (*“*He does what you do, so to speak*.”* [participant 5]), self-location (*“*Because you are controlling that body, so you are looking at it from the eyes of the virtual person*.”* [participant 15]), customization (*“*Because I just chose the same that I am*.”* [participant 3]), human-likeness, and haptics (*“*It looks real, and I also felt that I was touching my own hand.” [participant 24]). Throughout the iterations, ownership perceptions ranged from overall heterogenous to mostly positive; however, some participants remained ambivalent:

...Because I still know this in my real body and not that.

Participant 20

Regarding the teleport locomotion, participants reported heterogenous ownership feelings, illustrated by unrealistic movements and low agency, with a participant questioning the self-location after teleporting:

...Because you move forward so quickly I thought: “Will that body come with me.”

Participant 19

In contrast, joystick locomotion showed mostly positive ownership remarks, illustrated by the human-like movement illusions and agency through controller operation:

The movements I made with the joysticks, it made those too.

Participant 5

Furthermore, object manipulations showed positive ownership remarks owing to the gain of agency, manipulation realism, and human-likeness. In contrast, the perception of change decreased throughout the iterations. Although some participants in the second iteration felt lighter, smaller, or taller (“I was tall anyway, but I felt even taller when I was there in that game.” [participant 14]), participants in the third iteration reported only minor remarks (“My body just felt the same all the time.” [participant 20]). Notably, some participants disliked embodying an incongruent avatar (“I don’t want to be someone else.” [participant 18]), for example, in contexts of social interactions:

Because I think it’s important that I don’t mislead people.

Participant 15

**Table 2:** Extended Virtual Embodiment Questionnaire scores (sense of embodiment) related to iteration and task.

Context			Avatar customization, mean (SD)		Teleport locomotion, mean (SD)		Joystick locomotion, mean (SD)		Hybrid locomotion, mean (SD)		Object interaction, mean (SD)
**Sense of ownership**											
	Iteration 1	4.08 (1.69)		4.75 (2.41)		4.92 (2.04)		5.46 (2.28)		5.25 (2.32)	
	Iteration 2	5.15 (1.32)		4.83 (1.24)		5.62 (1.07)		5.81 (0.82)		5.96 (1)	
	Iteration 3	4.29 (1.16)		4.17 (1.72)		5.92 (1.19)		5.21 (1.44)		6.21 (0.95)	
	Overall	4.67 (1.41)		4.65 (1.65)		5.52 (1.38)		5.57 (1.40)		5.84 (1.41)	
**Sense of agency**											
	Iteration 1	5.71 (0.75)		5.71 (2.14)		6.08 (0.61)		5.96 (1.09)		5.96 (1.50)	
	Iteration 2	6 (1.19)		5.23 (1.35)		6.17 (0.86)		6.02 (0.98)		6.15 (1.01)	
	Iteration 3	5.67 (1.24)		5.46 (1.49)		5.96 (1.10)		5.38 (1.81)		6.38 (0.89)	
	Overall	5.84 (1.08)		5.41 (1.55)		6.09 (0.84)		5.84 (1.23)		6.16 (1.08)	
**Change (in the perceived body schema)**											
	Iteration 1	5 (1.59)		3.75 (2.24)		3.38 (2.22)		3.79 (2.33)		3.08 (2.78)	
	Iteration 2	3.65 (1.32)		3.06 (1.45)		3.42 (1.54)		2.85 (1.54)		2.56 (1.20)	
	Iteration 3	3.75 (1.69)		3 (1.74)		3.42 (2.06)		3.17 (2.08)		3.33 (2.04)	
	Overall	4.01 (1.53)		3.22 (1.69)		3.41 (1.77)		3.17 (1.84)		2.89 (1.84)	
**Sense of self-location**											
	Iteration 1	6.56 (0.66)		5.83 (1.01)		5.50 (1.66)		5.56 (2.33)		5.83 (1.76)	
	Iteration 2	6.44 (0.59)		6.22 (0.78)		6.14 (0.77)		6.42 (0.61)		6.22 (0.96)	
	Iteration 3	5.67 (0.97)		5.06 (1.83)		5.56 (1.03)		4.94 (1.85)		5.94 (1.04)	
	Overall	6.28 (0.77)		5.83 (1.22)		5.83 (1.10)		5.83 (1.58)		6.06 (1.17)	

### Locomotion Preferences and SoP

Most participants described a preference for joystick locomotion (15/24, 63%). Few participants selected the hybrid (5/24, 21%) or teleport locomotion (4/24, 17%). The latter was predominantly chosen by users who had experienced (severe) cybersickness. However, the dropouts (5/29, 17%) did not indicate their preference, which should be considered carefully.

The general presence (mean 6.12, SD 0.90) and spatial presence (mean 6.03, SD 0.81) were rated high compared with moderate involvement (mean 4.27, SD 1.88) and low realism (mean 3.59, SD 1.38) scores. The IVR environment was often described as unrealistic and boring; hence, participants suggested improving the graphics and realism, including some agents, objects (eg, chair, cars, and plants), or games, to make the experience more appealing. However, according to participant 11, this may cause overstimulation and distress.

## Discussion

### Principal Findings

This study reports on the feasibility and related design guidelines for IVBO in adults with MBID, by conducting a user-centered design approach with 3 iterations. In contrast to previous studies on IVR embodiment illusions, our avatar was tailored to the needs of our vulnerable group, by gradually adding interaction and customization abilities. In particular, we investigated the IVR avatar with related IK, controller-based locomotion, and (object) manipulation (Multimedia Appendix 2 [7,19,38,39,45,46,52,72,73]).

In the following sections, we discuss the findings related to our research questions. First, we discuss the feasibility to induce the illusion, influence of interactions on SoE, and guidance to enhance the immersion. Then, we discuss the design insights from our three IVR avatar components: (1) avatar appearance, (2) controller-based locomotion, and (3) object manipulation. Finally, we report on the limitations of our study, provide guidance for future studies, and provide a succinct summary of our contribution.

### Immersing People With MBID Into IVR Avatars

Our findings indicate that adults with MBID can embody anthropomorphistic IVR avatars from an egocentric perspective [27], even when avatar dimensions slightly differ from the self [24]. As expected, the highest ownership scores were achieved during object manipulation, requiring the amalgamation of interaction and navigation; however, adding locomotion that mimicked human walking was sufficient to enhance the IVBO compared with baseline [50]. Despite the participants’ desire to replicate their own body image through customization, this did not lead to effectual IVBO using our IVR. In contrast, adding body control was found to be decisive, suggesting that the sense of agency is vital for inducing ownership illusions in people with MBID [74-76]. This finding is further supported by a decreasing perception of change in the body schema during more extensive interactions, despite the unaltered avatar dimensions. However, the obtained sense of agency and self-location scores showed variance in active contexts, possibly owing to visuomotor incongruences or missing human-likeness during locomotion [28,50], occlusions during interaction [45], and extended insights into the limitations of the IK. Hence, we suggest further multisensory integrations [77], that is, advanced IK, animations, and physical interactions (eg, collision) with haptics to amplify the illusion [44,78]. Moreover, implementing more appealing IVEs could improve user involvement and realism, potentially enhancing IVBO [31,64]. However, using IVR avatars for people with MBID required extensive habituation periods when inducing and ending the IVBO, for example, by gradually adding control and providing support after acclimatization, as some participants described prolonged body sensations:

But for my own body I have to get used to it very much. Also when I take off the glasses, all at once bam, oh I’m here huh.

Participant 9

This process proved to be time consuming and complex, potentially hindering uptake and usability in practice. However, tailoring the IVR avatar to the user and use may circumvent this issue, allowing to integrate solely essential (and plausible) interactions, while considering user characteristics (eg, short attention and motor coordination issues) for a fitting immersion procedure.

### Designing IVR Avatars for People With MBID

The initial IVR avatar was developed based on the relevant literature and comprised models with high anthropomorphism from an egocentric perspective [38,72], customizable gender (man or woman) [73], skin tone [39], and body dimensions (ie, model and arms) [45,52]. In contrast to other studies, we omitted a mirror to inspect the virtual body, given that negative influences on ownership were suggested in previous studies [79]. Our results indicate that extended avatar customization could increase feelings of ownership for people with MBID, given the desire to replicate their own body image, in particular, the body dimensions. However, precise replication methods remain complex and are subject to future studies [39,80], limiting their applicability in consumer settings. However, replication fidelity in our design questions the need for personalization to induce IVBO in our target group. Instead, identification with the virtual body through a customization procedure seemed paramount, by replicating major body image characteristics with generic presets (*“*I’m really a slim puppet now. In real life I have a bit of a belly*.”* [participant 4]), as used in commercial social IVR applications (eg, Meta Horizon). Here, modifiable features (eg, clothing) appeared more trivial than body image features (eg, gender, skin tone, and corpulence). However, the body as reference frame could affect agency, interaction usability [45], and perception of the world [24,81], possibly resulting in unintended effects [23]. Furthermore, the lack of mirror and plain IVE could have reduced the incongruence awareness in our study [50], as IVR environments can influence perception [57], and facial properties may backfire when not personalized [39]. Nonetheless, our IK system proved sufficient to induce IVBO; however, avoiding impaired control was crucial, as occlusions showed more negative remarks than visual mismatches regarding leg movements [45,50]. Hence, functions for bending should be implemented to achieve sufficient proprioceptive congruence with the user’s body.

### Designing Controller-Based Locomotion Approaches for People With MBID

We investigated the design and user preferences for artificial IVR avatar locomotion approaches of continuous (ie, joystick) and noncontinuous nature (ie, teleport). Our findings in people with MBID indicate a favor for the joystick in contrast to the teleport or hybrid approach. This difference was explained by the human-likeness and fidelity during joystick locomotion, which can be supported by high ownership and agency values. In contrast to others, we considered user preferences during our design process, used approaches of different nature, and explored the effects on SoE [50]. Accordant with previous studies, natural walking animation was vital [50,53], and visuomotor incongruences between the model and stagnant user did not break the illusion [28,50,82]. Instead, users described a sense of agency via controller operation, which can be supported by the obtained SoE scores. Similar findings were observed in the study by Dewez et al [50], suggesting that control over the IVR avatar is paramount to visual congruence. A potential explanation for this walking illusion may be the user’s attention shift toward navigation, which reduces the awareness of visuomotor incongruences while providing a realistic movement illusion. In contrast to the similar SoE levels when comparing continuous techniques in populations that are not impaired [50], we found lower SoE scores when using the noncontinuous teleport. However, the prevalence of cybersickness and the resulting dropouts during joystick locomotion indicate severe usability drawbacks. Hence, designing for cybersickness alleviation seems essential to achieve both high SoE and usability, for instance, through adaptable locomotion. Our findings suggest tailoring FOV (eg, vignetting), turning (eg, snap turn and bodily turning), pace (eg, speed and range), and experience (eg, avoiding collision and stairs) to account for the needs of our group. In addition, enabling control habituation was crucial, given that artificial approaches tend to increase the cognitive workload [46]. Finally, hybrid locomotion was redundant because users relied on their preferred technique. However, it remains interesting to explore this approach in more experienced users, as it allows fast movement without cybersickness, while providing fidelity for object manipulation.

### Designing Controller-Based (Object) Manipulations for People With MBID

We further explored the controller-based IVR avatar interaction by allowing users to engage in object manipulation tasks with the customized body and preferred locomotion approach. Here, designing for an intuitive interaction was decisive, with realistic animations of virtual hands, physical collisions, and related haptics, further supporting the suggested multisensory integration to enhance IVBOs. Although not implemented in our prototype, tailored hand animations could be used to avoid visual interpenetration with virtual objects [45]. Previous study has shown that users favor defined hand poses [83]; however, constraints through limited animations could reduce SoE and affect performance [45]. For interactions with objects that are placed low, implementing a spherecast that is *magnetic* outperformed raycasting and bending. Although bending was attempted intuitively by our participants, it resulted in severe balance errors that can potentially cause injury. In contrast, raycasting showed severe usability drawbacks for small objects, presumably because the visual and haptic feedback was merely activated when hitting the object. Notably, using artificial interactions did not entail negative remarks, presuming that the control remained unimpaired. Nonetheless, object interaction using full IVBO is understudied, particularly when combined with artificial locomotion. During our design process for people with MBID, we observed usability issues when both were combined. Although control habituation could reduce these issues, providing a generous room-scale area for object manipulation seemed more user-friendly; however, physical walking was limited to 2×2 m. Nonetheless, as space is mostly restricted, we encourage others to further explore the requirements for an unobtrusive amalgamation of interaction and locomotion. Finally, the operation of object-spaced and screen-spaced UIs showed good usability with no negative effects, indicating the potential for autonomous use of such IVR applications by our target users.

### Limitations and Future Studies

Our study has some limitations that should be considered. First, our convenience sampling in the design process included mainly male participants with some technology literacy, which may reduce the generalizability of the findings to the diverse group with MBID. Second, we failed to achieve an accurate scaling method in the first 2 iterations owing to technical issues, considering that the state-of-the-art system was still in the beta stage and applied outside the controlled laboratory setting. However, the findings provide valuable insights for our design and hint toward applications of implicit learning (eg, proteus effect) [23], as SoE was observed despite inaccurate avatar dimensions. Third, severe cybersickness issues resulted in dropouts, which may have biased the obtained data, such as locomotion preferences. Fourth, questionnaires were assessed verbally, possibly leading to an increased social desirability bias, whereas paper-based approaches can increase complexity. Previous study from our group suggests that a Visual Analogue Scale implemented in IVR may be more appropriate [7]. Finally, we used a plain IVE that may reduce spatial awareness, such as height and object size perception [24]. Hence, we suggest using spatial cues in future studies, which should be carefully selected to avoid distress (ie, overstimulation) in people with MBID.

Future studies should build upon our findings to further refine our guidelines for IVR avatars for people with MBID to design natural IVR interactions and learning (eg, psychotherapy, health education, and life skills training). Here, influences on SoE should be investigated to evaluate the interaction design and confirm the feasibility of IVBO in diverse samples (eg, technology literacy and intellectual and adaptive functioning). From a technical standpoint, exploring multisensory integrations (eg, advanced IK, interaction animations, haptics, and physics-based manipulations) appears to be paramount to enhance the feeling of agency, as natural and unimpaired interactions seem to be pivotal for IVBO. However, investigating advanced body replication methods as opposed to more generic presets seems to be important to understand the self-attribution to IVR avatars in people with MBID. Our prototyping in the care setting revealed that customization and habituation procedures were complex and tedious, potentially hindering the applicability of IVBO in people with MBID. Hence, using a balanced design by conducting habituation periods (ie, adapting locomotion and interaction) before avatar customization seems promising to reduce the required time for inducing IVBO. This implies neglecting properties that are more trivial in the given use context, such as clothing or facial features, which may be more relevant in social or collaborative IVR. Furthermore, the application of our locomotion and manipulation modules should be investigated with varying degrees of embodiment (eg, full body vs hands) to tailor the interaction design to the individual user and use case. This could reduce the tailoring effort and occurrence of adverse effects (eg, cybersickness), for instance, by limiting locomotion to room scale for body swapping scenarios, whereas public transport trainings may profit from artificial techniques for an extended range. For cueing, using game design and narratives seems promising, as common visual interaction cues (eg, light beams, leading lines, and placement cues) and aids (eg, vantage points and landmarks) showed adequate usability. Finally, we combined promising design components; however, a plethora of other interaction techniques can be explored, such as redirected walking to further alleviate cybersickness.

### Conclusions

Our findings suggest that adults with MBID can embody gender-matched IVR avatars with high anthropomorphism. To induce IVBO, having a high sense of agency over the virtual body appeared to be crucial, ideally with corresponding multisensory feedback, such as physics-based collisions and haptics. This is consistent with previous studies on place illusion and plausibility illusion [16], suggesting that plausible interactions are vital for IVBO in our group. However, implementing artificial aids into the virtual body (ie, spherecasting and raycasting) was not perceived as disruptive, presuming that the control was not impaired. Customizing the avatar according to the participant’s body image appeared to boost the illusion; however, it was complex and tedious, affecting the practicability of IVBO, as individuals with MBID showed an extensive need for (control) habituation. Therefore, balancing IVBO immersion by focusing on habituation and lowering customization effort seems to be crucial to achieve both high SoE and usability. Owing to the limited attention span of people with MBID, tailoring to user and use appears to be important. Considering the cognitive limitations, we advise to avoid artificial interaction techniques that are implausible and increase the cognitive workload (eg, teleport) or evoke severe side effects, if possible, for the intended use context. In contrast, the use of artificial techniques comes at the expense of learning time and cognitive load, possibly interfering with other immersion parts. In conclusion, although designing IVR avatars for people with MBID is not fundamentally different, users’ limitations challenge designers to develop tailored immersion procedures. Future studies should further investigate guidelines for IVR avatars in people with MBID by designing natural interactions, including multisensory integrations and other interaction approaches (eg, hand tracking and redirected walking). In addition, procedures and use cases for implicit and explicit learning should be explored, for instance, as a tool for playful health behavior change interventions. For this, the necessity of interactions should be reviewed carefully to avoid adverse effects (eg, cybersickness) and reduce the burden when interacting with IVR for people with MBID.

## Appendix

Multimedia Appendix 1 Semi-structured interview –IVR avatars for embodiment illusions in people with MBID.

Multimedia Appendix 2 Guidelines for designing IVR avatars for embodiment illusions in people with MBID.

## Abbreviations

FOV: field of view
HMD: head-mounted display
IK: inverse kinematics
IPQ: Igroup Presence Questionnaire
IVBO: illusions of virtual body ownership
IVE: immersive virtual environment
IVR: immersive virtual reality
MBID: mild to borderline intellectual disability
POV: point of view
SoE: sense of embodiment
SoP: sense of presence
UI: user interface
VEQ: Virtual Embodiment Questionnaire
