# Supplementary material for: Immersive Virtual Reality Avatars for Embodiment Illusions in People With Mild to Borderline Intellectual Disability: User-Centered Development and Feasibility Study
Source: JMIR Serious Games. 2022 Dec 7;10(4):e39966. doi: 10.2196/39966 (PMC9773028; doi:10.2196/39966)
Supplement: Multimedia Appendix 1 [file games_v10i4e39966_app1.docx]

**Appendix**

**Appendix 1.** Semi-structured interview –IVR avatars for embodiment illusions in people with MBID.

| T1: Avatar (+ IK): | | Additional: |
| --- | --- | --- |
| AV1 | Wat vond je van de avatar?  (What did you think of the avatar?) |  |
| AV2 | Wat vond je van het veranderen van de grote van de avatar?  (What did you think about changing the size of the avatar?) |  |
| AV3 | Heb je je eigen geslacht gekozen voor de avatar?  (Did you choose your own gender for the avatar?) | Waarom?  (Why?) |
| AV4 | Heb je je eigen huiskleur gekozen voor de avatar?  (Did you choose you own skin tone for the avatar?) | Waarom?  (Why?) |
| AV5 | Had je het gevoel dat het virtuele lichaam van jou was?  (Did you feel like the virtual body was your own?) | Waarom?  (Why?) |
| AV6 | Wat denk je wat er verbeterd kan worden?  (What do you think can be improved?) |  |
| T2-4: Avatar + locomotion (Joystick, teleport, or hybrid): | | Additional: |
| AL1 | Wat vond je van de bewegingstechniek?  (What did you think of the locomotion technique?) |  |
| AL2 | Welke problemen ben je tegengekomen bij het bewegen in de virtuele wereld?  (What problems did you encounter when moving in the virtual world?) |  |
| AL3 | Wat vond je van de avatar tijdens het bewegen?  (What did you think of the avatar when moving?) |  |
| AL4* | Had je het gevoel dat het virtueel lichaam van jou was?  (Did you feel like the virtual body was your own?) | Waarom?  (Why?) |
| AL5 | Heb je nog andere opmerkingen?  (Do you have additional remarks?) | Welke?  (Which?) |
| T5: Avatar + preferred locomotion + interaction: | | Additional: |
| AI1 | Hoe lukte het met dingen in de virtuele wereld om te gaan?  (How did you manage to interact with things in the virtual world?) | Waarom? (Why?) |
| AI2 | Welke problemen had je om met dingen om te gaan?  (Which problems did you encounter when interacting with things?) |  |
| AI3 | Wat vond je leuk aan de interactie met objecten?  (Wat did you like when interacting with objects?) |  |
| AI4 | Wat vond je van de avatar tijdens omgaan met objecten?  (What did you think about the avatar when interacting with objects?) |  |
| AI5* | Had je het gevoel dat het virtuele lichaam van jou was?  (Did you feel like the virtual body was your own?) | Waarom?  (Why?) |
| AI6 | Heb je nog andere opmerkingen?  (Do you have additional remarks?) | Welke?  (Which?) |
| T6: Post-experiment: | | Additional: |
| PE1 | Wat vond je van de oefenomgeving?  (What did you think about the training environment?) |  |
| PE2 | Welke problemen heb je gehad in de oefenomgeving?  (Which problems did you encounter in the training environment?) |  |
| PE3* | Mensen kunnen het gevoel krijgen dat het eigen lichaam anders voelt in VR. Hoe was het met jou?  (People may feel that their own bodies feel different in VR. How was it for you?) | Vorm, gewicht, lengte, breedte?  (Form, weight, length, width?) |
| PE4 | Hoe lukte het instellingen aan te passen (zoals de bewegingstechniek of het interactiespel)?  (How did you manage to adjust settings (such as the movement technique or the interaction game)?) | Waarom?  (Why?) |
| PE5 | Hoe lukte het om te teleporteren naar verschillende locaties (A indrukken en selecteren)  (How did you manage to teleport to different locations (press A and select)?) | Waarom?  (Why?) |
| PE6 | Aan wie zou je deze activiteit aanraden?  (To whom would you recommend this activity?) |  |
| PE7 | Heb je nog andere opmerkingen?  (Do you have additional remarks?) | Welke?  (Which?) |

* Items added after iteration 1
