# Supplementary material for: Immersive Virtual Reality Avatars for Embodiment Illusions in People With Mild to Borderline Intellectual Disability: User-Centered Development and Feasibility Study
Source: JMIR Serious Games. 2022 Dec 7;10(4):e39966. doi: 10.2196/39966 (PMC9773028; doi:10.2196/39966)
Supplement: Multimedia Appendix 2 [file games_v10i4e39966_app2.docx]

**Appendix 2.** *Guidelines for designing IVR avatars for embodiment illusions in people with MBID.*

| **Component** | **Recommendations** |
| --- | --- |
| 1. Avatar (IK) | - 1. Implement an anthropomorphistic 3D model that acts as self-representation in IVR environments [38, 77].   2. Implement customization presets to allow replicating the major body image characteristics, i.e. gender, skin-tone, and body dimensions [39, 45, 52, 78].   3. Implement an IK-system (based on three-point tracking) with suitable bending functions, i.e. upper body and legs, to increase proprioception and avoid occlusion.   4. Consider including physical properties, i.e. collision with haptics (e.g. self-touch) and weight, to enhanced multisensory experiences [19]. |
| 1. Artificial locomotion | - 1. Implement joystick locomotion with corresponding walking animation as default technique to induce the IVBO [46].   2. Implement teleport (with room-scale area) locomotion as alternative for individuals affected by cybersickness [46].   3. Consider adapting the vision (e.g. vignetting), turning (e.g. snap-turn, room-scale), pace (e.g. speed, range), experience (e.g. stairs, collision) to reduce locomotion-induced cybersickness. |
| 1. Interaction | - 1. Implement hand animations tailored to the object to avoid occlusions [45].   2. Implement a (‘magnetic’) spherecast to grab objects from lower areas.   3. Implement an adequate room-scale area for interaction-related repositioning.   4. Implement object-spaced and screen-spaced (fix on Y-axis) UI interactions for autonomous usage of such systems, by integrating raycasting techniques into the virtual hands.   5. Consider including physical properties, i.e. accurate collision with haptics, to enhance congruent multisensory experiences during interaction [45]. |
| 1. Procedural | - 1. Implement habituation procedures to learn controls and induce the IVBO.   2. Implement a generic scaling of the model and further customization with presets to avoid prolonged habituation periods, by reducing the customization effort to a minimum.   3. Implement post-acclimatization support (with care staff).   4. Consider adding visual in-game instructions to support the user-researcher communication when using an asymmetric setup.   5. Consider using more realistic environments with spatial cues during research, though avoiding distress for the user (e.g. many agents) seems crucial.   6. Consider implementing a single-item VAS in IVR instead of asking standardized questionnaires to account for the needs of people with MBID [7]. |

Abbreviations: IVR = Immersive Virtual Reality, IK = Inverse Kinematics, IVBO = Illusion of Virtual Body Ownership, UI = User Interface, VAS = Visual Analogue Scale, MBID = Mild to Borderline Intellectual Disability.
